# Depth-Sensing Hardness Measurements to Probe Hardening Behaviour and Dynamic Strain Ageing Effects of Iron during Tensile Pre-Deformation

**DOI:** 10.3390/nano11010071

**Published:** 2020-12-30

**Authors:** Lyubomira Veleva, Peter Hähner, Andrii Dubinko, Tymofii Khvan, Dmitry Terentyev, Ana Ruiz-Moreno

**Affiliations:** 1 European Commission, Joint Research Centre, Directorate G: Nuclear Safety and Security, Westerduinweg 3, 1755 LE Petten, The Netherlands; lyubomiraveleva@gmail.com (L.V.); ana.ruiz-moreno@ec.europa.eu (A.R.-M.); 2 SCK•CEN, Nuclear Materials Science Institute, Boeretang 200, 2400 Mol, Belgium; andrii.dubinko@sckcen.be (A.D.); tymofii.khvan@sckcen.be (T.K.); dmitry.terentyev@sckcen.be (D.T.)

**Keywords:** nanoindentation, unalloyed iron, strain hardening, atomic force microscopy, pile-up, dynamic strain ageing

## Abstract

This work reports results from quasi-static nanoindentation measurements of iron, in the un-strained state and subjected to 15% tensile pre-straining at room temperature, 125 °C and 300 °C, in order to extract room temperature hardness and elastic modulus as a function of indentation depth. The material is found to exhibit increased disposition for pile-up formation due to the pre-straining, affecting the evaluation of the mechanical properties of the material. Nanoindentation data obtained with and without pre-straining are compared with bulk tensile properties derived from the tensile pre-straining tests at various temperatures. A significant mismatch between the hardness of the material and the tensile test results is observed and attributed to increased pile-up behaviour of the material after pre-straining, as evidenced by atomic force microscopy. The observations can be quantitatively reconciled by an elastic modulus correction applied to the nanoindentation data, and the remaining discrepancies explained by taking into account that strain hardening behaviour and nano-hardness results are closely affected by dynamic strain ageing caused by carbon interstitial impurities, which is clearly manifested at the intermediate temperature of 125 °C.

## 1. Introduction

Nanoindentation constitutes one of the micromechanical testing techniques used to study mechanical properties at small scales and/or of small specimens. At these scales, the understanding of the dislocation mechanisms acting during indentation and their interaction with the specimen’s surface is key to comprehending the mechanical response of the material [1,2,3]. Continuous improvements in methodologies to record and analyse load—displacement curves are promoting nanoindentation to complement conventional mechanical testing in materials selection and design [4]. Furthermore, the technique allows probing of positions with varying properties, for instance, across the thickness of a larger component. While the measured indentation hardness depends on the material properties, like elastic modulus, yield stress and strain hardening behaviour, it is also affected by the indenter geometry and size.

The attractiveness of nanoindentation as a mechanical test technique relates to the convenience of providing large amount of data with a comparatively low amount of effort and time. On the downside, nanoindentation data are affected by the indentation size effect (ISE) [5,6,7], which has to be determined in order to compare with mechanical properties at a larger scale. Moreover, the formation of pile-ups of material around the indenter mark can take various degrees and largely affect the apparent hardness, thereby invalidating nanoindentation for quantitative mechanical property assessment, unless the technique is complemented by costly atomic force microscopy (AFM). It is important to note that nano-scale physical processes related to the dislocation motion, self-pinning and multiplication and their interactions with grain boundaries drive the formation of the pile-ups. The operation of these nano-scale mechanisms may lead to the accumulation of considerable and non-negligible plastic deformation depending on the initial microstructure (i.e., dislocations, point defects impurities, grain boundaries) and ability for plastic deformation (Peierls stress, crystallographic structure).

This work reports results from quasi-static nanoindentation measurements of unalloyed iron at room temperature, performed in order to extract hardness and elastic modulus as functions of indentation depth (size effect), as well as to assess the effect of pre-deformation. This assessment turns out to be complicated by the pile-up formation during nanoindentation, because an increased disposition for pile-up formation [8] of the iron following pre-straining is observed, which leads to significant increases of the apparent hardness and indentation modulus levels.

As the present investigation uses nanoindentation for mechanical property correlation, grain boundaries can be expected to affect load–displacement curves, indentation Young’s moduli, von Mises stresses, and pile-up formation, see for instance the work of Liu et al. [9] where this was studied by crystal plasticity finite element simulation of bicrystal deformation during nanoindentation. Pile-up behaviour is also anisotropic due to the activation of different slip systems and, hence, pile-up patterns depend on the grain orientations as demonstrated through crystal plasticity modelling in ref. [10]. Under these circumstances, nanoindentation can either be complemented by grain orientation information from electron backscatter diffraction (EBSD) or like in the present work, by averaging over multiple grains with random orientations. To relate nanohardness data to tensile properties, constraint effects [11], ISE [12], and grain size effects [13] can be taken into account.

The effects of pile-up emergence on the mechanical properties of the material have therefore been investigated, in order to derive meaningful hardness results, which are adjusted for artefacts from the pile-up formation. Strain hardening behaviour and nanoindentation hardness results are interpreted in relation to dynamic strain ageing (DSA) behaviour of the material [14,15].

## 2. Materials and Methods

### 2.1. Material

The unalloyed Fe cast material used for this study was produced by OCAS NV in Belgium in an induction vacuum furnace and designated as G379. A piece from the produced material was introduced in a pre-heated furnace at 1200 °C for 1 h and hot rolled without interruption. The as-received sheet was subsequently air-cooled to room temperature. The final dimensions of the sheet were approx. 10 mm (height) × 250 mm (width) × 600 mm (length). The average grain size of the material was determined as 86 μm by linear intercept method applied to an EBSD map of the material.

The chemical composition in the middle of a slice cut from between the head and the body of the ingot was determined by OCAS NV by means of spark source optical emission spectroscopy (SS-OES) to quantify all elements except Ni, Si and Al, and by inductively coupled plasma optical emission spectroscopy (ICP-OES) giving access to Ni, Si and Al concentrations. The results are shown in Table 1; all other elements were below the SS-OES detection limits, except for the S content, which was between 10 and 15 ppm for this cast. It is important to point out that a C impurity content of 0.0067 wt.% would correspond to a concentration of 313 ppm, which is significant in inducing dynamic strain ageing [15].

### 2.2. Mechanical Deformation of Specimens

G379 Fe specimens were pre-strained to 15% by applying uniaxial tensile deformation. The mechanical straining was performed on an Instron electro-mechanical universal test machine (Instron, Norwood, MA, USA) equipped with an environmental chamber and subject to regular qualification and calibration procedures according to Belgian accreditation rules (BELAC). Sample elongation was measured by the pull rod displacement. The force was measured by a load cell with a maximum capacity of 50 kN. Uniaxial tensile tests at a crosshead displacement rate of 0.2 mm/min were performed on flat dog-bone shaped specimens featuring a gauge length of 60 mm and a cross-section of 1.1 × 12 mm^2^. The overall length of the specimens was 120 mm. The tests were carried out at room temperature, 125 °C and 300 °C in air up to 15% deformation. The plastic deformation was uniform across the whole gauge section as confirmed by the dimensional check after the test.

### 2.3. Microstructural Characterization

The microstructure of as-received and plastically deformed samples was studied by transmission electron microscopy (TEM) to provide information on the dislocation density and possible grain refinement caused by the plastic deformation. The pieces for TEM samples were mechanically polished from both sides using SiC paper with grit sizes of 220, 500, 1200 and 4000 to achieve 70–100 μm thickness and further cut with a wire cutter into pieces to fit 3 mm TEM grids. They were polished again from both sides with 4000 SiC paper to remove the remnants of a glue, rinsed in acetone and ethanol and then glued on 3 mm copper grids with an aperture of 1 mm. Finally, TEM specimens were polished electrochemically with a solution of 1.5 wt.% NaOH in water with an applied voltage of 30 V.

The specimens were investigated with a JEOL 3010 TEM (JEOL Ltd., Tokyo, Japan) operating at 300 kV. The average dislocation density was measured following the methodology used in [16,17,18]. Each calculation requires a TEM micrograph, the corresponding diffraction pattern and a convergent beam electron diffraction (CBED) pattern. Several calculations at different areas of the specimen were performed to get an average dislocation density. In the software DigitalMicrograph (Gatan Inc., Pleasanton, CA, USA), provided with the image sensor of a microscope, a circle is drawn randomly in an image and the number of intersections of it with dislocation lines is counted. The dislocation density is then calculated as *ρ* = 2*N/Lt*, where *N* is the number of intersections of the circle with dislocation lines, *L* the perimeter of the circle, *t* the local thickness of the specimen at the area of the image. The perimeter is automatically calculated by the software, while the local thickness of the specimen is determined from the CBED pattern and the diffraction pattern.

**Figure 1:**
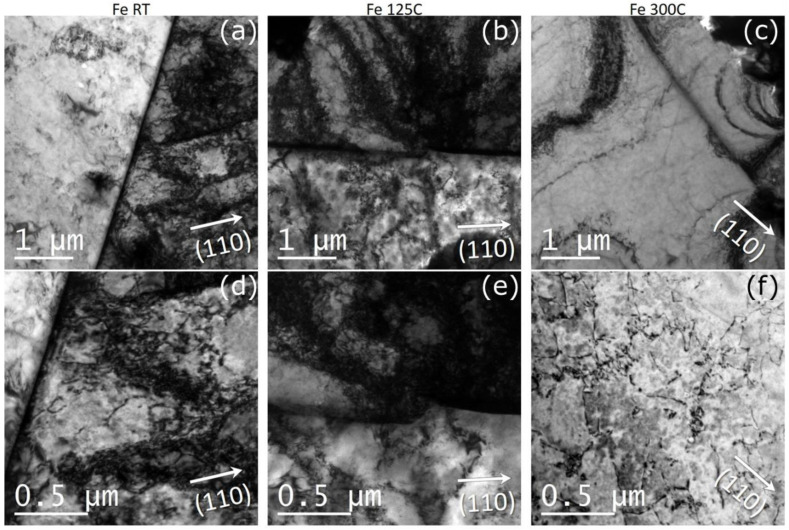
TEM micrographs showing typical dislocation patterns in plastically deformed Fe. (**a**,**d**)—deformed at room temperature, (**b**,**e**) deformed 125 °C, (**c**,**f**) deformed at 300 °C.

Patterns of the dislocation microstructure after the plastic deformation are provided in Figure 1. In the non-deformed samples, the dislocations are homogeneously distributed, and their density is about 10^12^ m^−2^. In the pre-deformed samples, the dislocations appear to be heterogeneously distributed for all deformation temperatures, i.e., some regions exhibit high density with dislocation pile-ups and tangles, whereas other regions contain rather low dislocation densities of about 10^12^ m^−2^, comparable to the density in the non-deformed material. Following the method described above, the mean density was determined to amount to 2 × 10^14^ m^−2^, 3.7 × 10^14^ m^−2^ and 8 × 10^13^ m^−2^, for samples deformed to 15% of strain at RT, 125 °C and 300 °C, respectively.

### 2.4. Nanoindentation Testing

For nanoindentation testing and AFM of the indents, three samples of 10 × 10 × 1 mm^3^ were cut from the gauge length (deformed part of the specimen) and three other samples were taken from the shoulder (not deformed) of the tensile specimens, as described in Table 2.

The sample surface preparation sequence is summarized in Table 3**.** The surface was first ground with abrasive paper with grit 320 (SiC), followed by grinding with grit 800, then mechanically polished with 6 μm, 3 μm and final polishing with 1 μm diamond paste.

Indentation tests were performed using a high temperature Ultra nanoindentation test device UNHT HTV (Anton Paar GmbH, Graz, Austria) with dual indenters (indentation and active reference tip) that provides active surface referencing and thereby minimizes drift and frame compliance. A Berkovich diamond self-similar tip was used for the indentation tests. The tip area function and the frame compliance were calibrated according to ISO 14577-2 using a DataSure IIT kit of certified reference materials (tungsten and fused silica) from National Physical Laboratory in an iterative way [19].

Force-controlled single cycle (FSC) measurements were carried out with linear loading up to various maximum forces *F*_max_ and concomitant contact depths *h*_c_ with a loading time of 30 s, a dwell time of 10 s, and subsequent unloading within 30 s. Five maximum force levels of 1 mN, 5 mN, 10 mN, 50 mN and 100 mN were applied. For each condition, ten repeat indents were randomly placed over the surface of the material and the results averaged. Random positioning of indents was achieved by spacing them equidistantly 50 μm apart in a square lattice irrespective of the underlying polycrystalline grain structure. The lattice spacing was chosen with a view to the grain size and to avoid mutual interaction of even the largest indentations.

**Figure 2:**
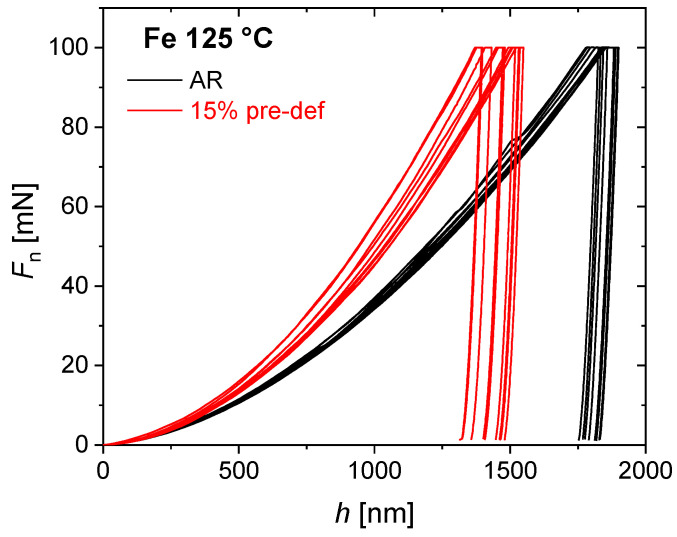
Indentation force *vs.* depth curves for two sets of repeated indents to 100 mN maximum force on the Fe-AR-125 and the Fe-15%-125 specimen, respectively.

**Figure 3:**
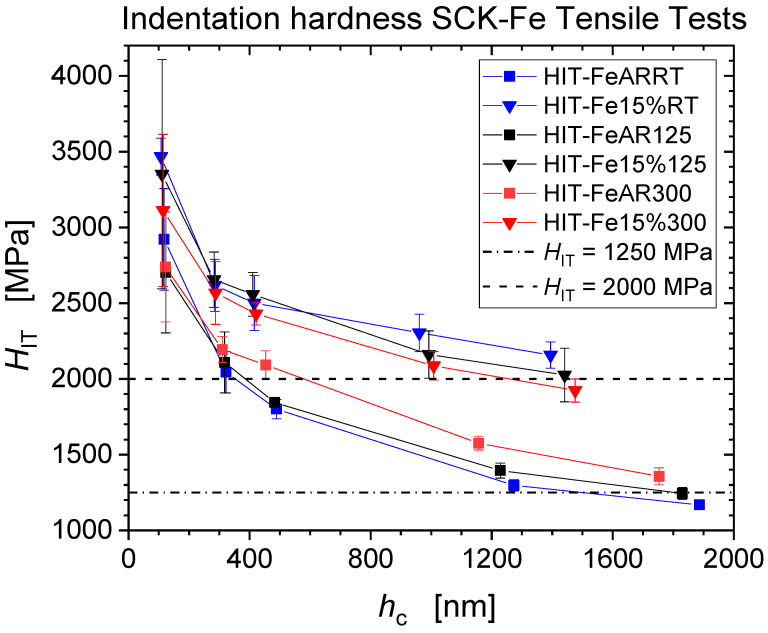
Depth dependence of hardness of un-strained and 15% pre-strained Fe from nanoindentation measurements.

Figure 2 illustrates the indentation force vs. depth curves measured for two sets of repeated indentations to 100 mN maximum force on the Fe-AR-125 and the Fe-15%-125 specimen, respectively. One notes good repeatability as plastic anisotropy of the material is low, noting that the hardness spread increases significantly after pre-deformation. This effect is most pronounced for the intermediate temperature of 125 °C illustrated here, cf. Figure 3.

The measured data were analysed according to ISO-14577 by fitting the 98% to 40% *F*_max_ portion of the unloading curve to a power law and using the fitted parameters to calculate the contact depth *h*_c_ and stiffness *S*. Indentation hardness *H*_IT_ and reduced modulus of the contact *E*_r_ are then determined by the calibrated projected area of the contact between the indenter and the sample *A*_p_:

(1) $$H_{\mathrm{IT}}=\frac{F_{\max}}{A_{p}\left(h_{c}\right)}$$

(2) $$E_{r}=\frac{\sqrt{\pi }S}{2\beta \sqrt{A_{p}\left(h_{c}\right)}}$$

where *β* is a geometric factor, which amounts to 1.034 for a Berkovich indenter. The indentation modulus relates to the reduced modulus according to

(3) $$E_{\mathrm{IT}}=\frac{1-\nu_{s}^{2}}{\frac{1}{E_{r}}\;-\;\frac{1\;-\;\nu_{i}^{2}}{E_{i}}}$$

with Poisson ratios of the Fe samples and the diamond indenter *ν*_s_ = 0.3 and *ν*_i_ = 0.07, respectively, and *E*_i_ = 1141 GPa for the elastic modulus of the indenter.

After the nanoindentation tests, indents were imaged by AFM with a Nanosurf, Switzerland equipped with pyramidal silicon ACLA tip, for non-contact tapping mode, and using dynamic force mode.

## 3. Results and Discussion

### 3.1. Hardness and Young’s Modulus from Nanoindentation

**Figure 4:**
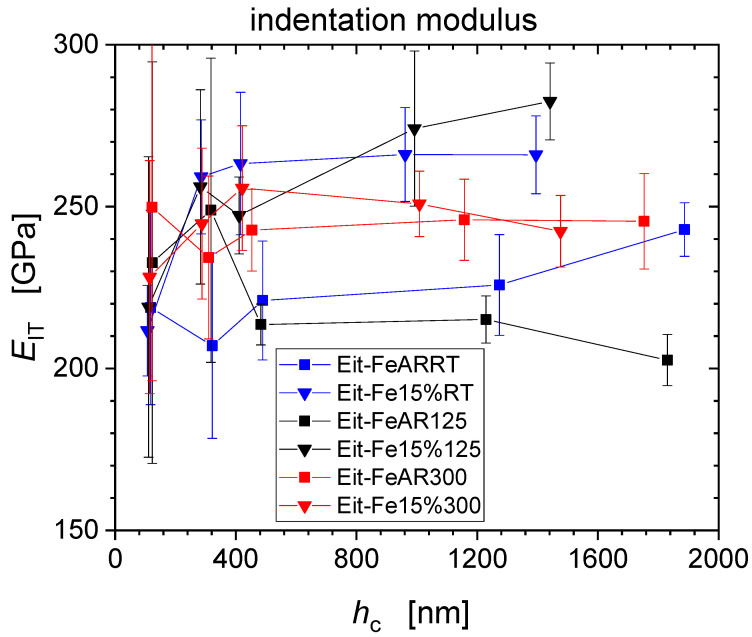
Indentation modulus values of un-strained and 15% pre-strained Fe as measured by nanoindentation.

Figure 3 and Figure 4 present the experimental results of the averaged indentation hardness (*H*_IT_) and the indentation elastic modulus (*E*_IT_) from the force-controlled nanoindentation measurements at the five force levels *F*_max_ *=* {1; 5; 10; 50; 100} mN for the as received, and the pre-strained iron. Error bars correspond to the standard deviations from the 10 repeat measurements for each condition. A pronounced indentation size effect (ISE) is visible for all specimens [6,7,12]. As one compares the data from as-received and pre-strained Fe for the three tensile deformation temperatures RT, 125 °C and 300 °C, one observes that the pre-strained conditions level out at micro-hardness values of about 2000 MPa, significantly higher than the as-received conditions at about 1250 MPa. Indentation modulus values do not show significant depth dependence but exhibit larger levels of scatter as compared to hardness values ranging from ~190 GPa to ~240 GPa for the as-received material, while moduli tend to increase for the pre-strained conditions ranging from ~250 GPa up to ~300 GPa. This is an indication for increased disposition for pile-up formation following pre-straining of the material [8].

The appearance and morphology of pile-ups depend on the work hardening of the material [20]. The hardness is inversely proportional to the contact area and the Young’s modulus is inversely proportional to the square root of the contact area [21]. Since the Oliver-Pharr method used for evaluation is based on the contact area as calibrated with respect to the plane of the original surface, rather than the actual contact area receiving the load [8,22,23], the contact area is underestimated in the presence of the pile-up formation causing the values of hardness and reduced modulus to be overestimated.

### 3.2. Pile-Up Effect on the Mechanical Properties

**Figure 5:**
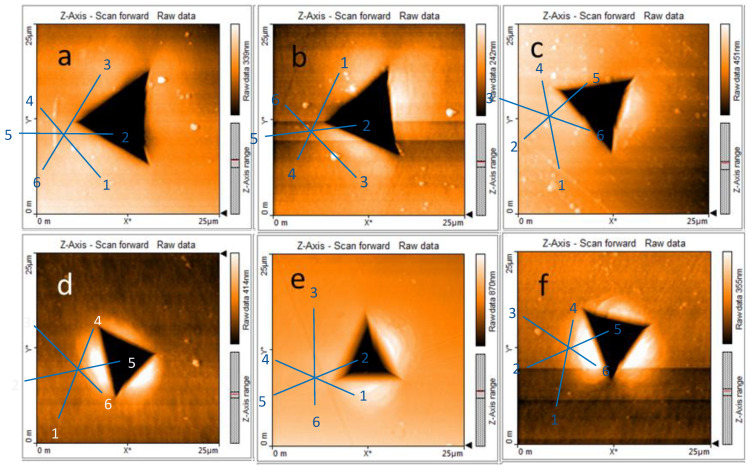
AFM images of Fe after nanoindentation: (**a**) Fe-AR-RT, (**b**) Fe-AR-125 °C, (**c**) Fe-AR-300 °C, (**d**) Fe-15%-RT, (**e**) Fe-15%-125 °C, (**f**) Fe-15%-300 °C, with the directions numbered as shown for the depth profiles of Figure 6.

**Figure 6:**
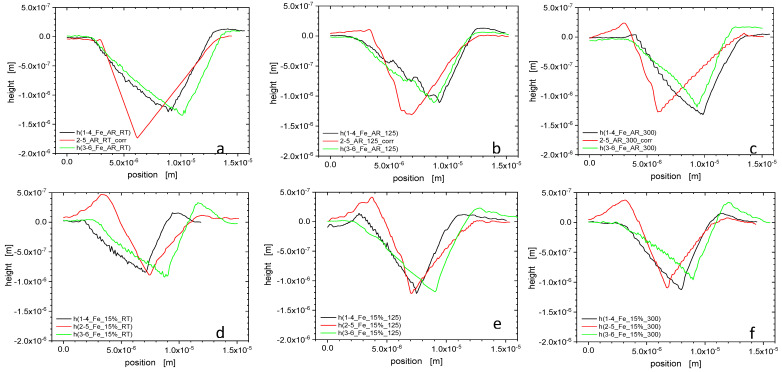
Cross-sectional height profiles from AFM images of Fe after nanoindentation: (**a**) Fe-AR-RT, (**b**) Fe-AR-125 °C, (**c**) Fe-AR-300 °C, (**d**) Fe-15%-RT, (**e**) Fe-15%-125 °C, (**f**) Fe-15%-300 °C, cf. previous figure. The 2–5 profiles of Figure 6a–c have been corrected for zero reference height away from the indent. From the AFM images and the resulting cross-sectional profiles, one notes that the formation of pile-ups in the non-deformed state of the material is relatively weak as compared to the significant piling-up observed in the pre-deformed state of the material. Pre-deformation enhances the pile-up propensity, which affects the apparent nanoindentation hardness.

Significant pile-up behaviour as anticipated from nanoindentation results has been confirmed by AFM measurements. Figure 5 presents AFM images (25 μm × 25 μm) obtained from as-received and pre-deformed Fe samples after nanoindentation with 100 mN force. To quantify the developed pile-ups, cross-section profiles corresponding to the AFM images obtained from the lines crossing the three distinct sides of the indentation edges have been determined (Figure 6). Due to the geometry of the Berkovich indenter, the pile-up heights at the corners of the triangular impression are small as compared to those adjacent to the side edges. For each indent, three cross-sectional profiles have been acquired and pile-up heights measured as the difference in height between the material upheaval peak and the original sample surface ahead of each corner or side [23].

**Figure 7:**
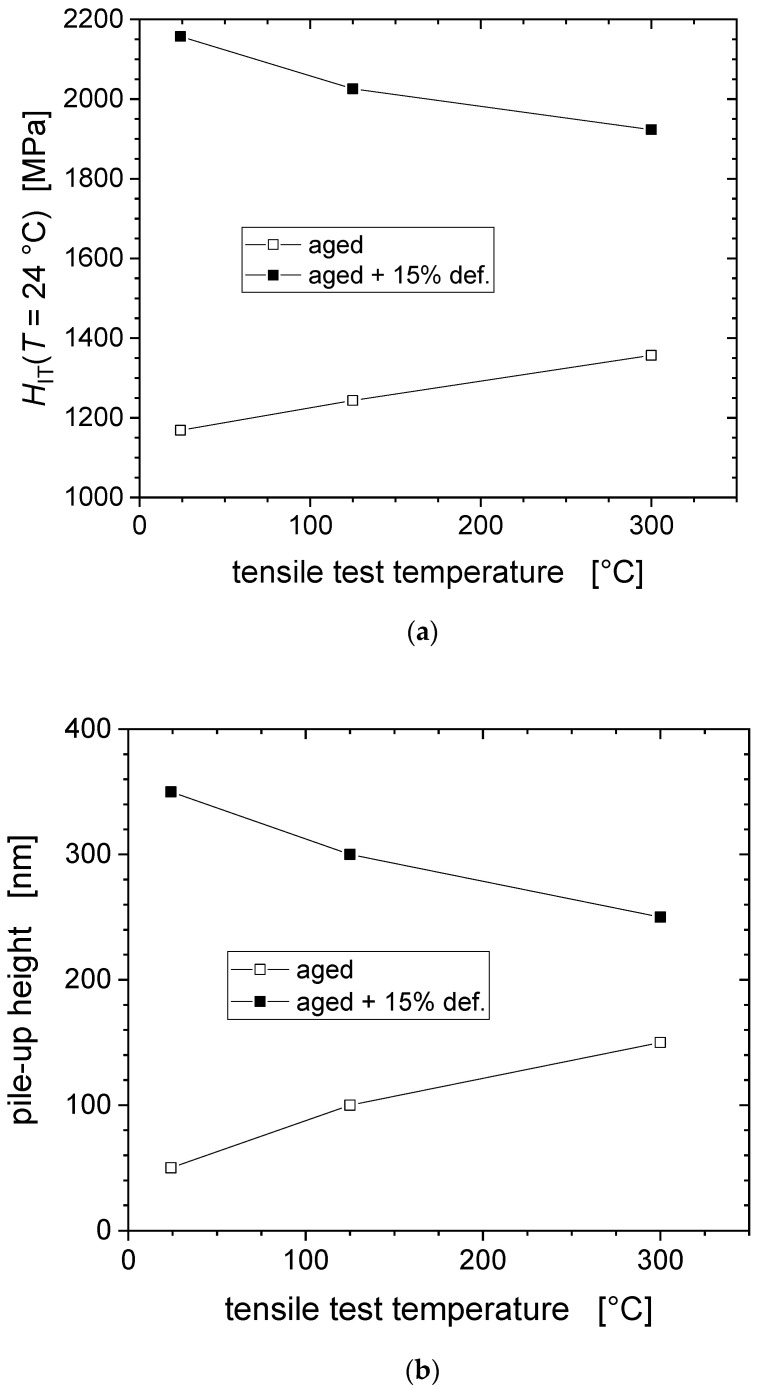
Correlation between the indentation hardness measured at RT and the tensile test temperature (**a**), and between pile-up effect and tensile test temperature (**b**).

Figure 7 reveal the relation between hardness and pile-up height, respectively, as a function of tensile test temperature, and the effect that the pre-deformation has on it. The pile-up heights from room temperature indentation for the non-deformed states increase with increasing tensile pre-deformation temperature, i.e., ageing temperature, unlike what holds for the pre-deformed state of the material, where the hardness and the pile-up heights decrease with increasing tensile test temperature, i.e., pre-deformation temperature. The temperature and pre-deformation dependence of the hardness are closely associated with the pile-up height. The increased propensity for pile-up formation of the pre-deformed state is attributed to a lower strain hardening rate of the plastically pre-deformed material [8].

### 3.3. Relation between Mechanical Properties from Tensile Tests and Hardness Measurements

Self-similar pyramidal indenters like Berkovich indenters induce plastic deformation already for the smallest loads when tip blunting is disregarded. Therefore, yielding as the onset of plastic deformation of the material is not as straightforward to access as in the analysis of a tensile test. A geometrically perfect self-similar Berkovich indenter is more properly associated with a finite representative strain.

Tabor has argued that the tensile strength of a material affects its hardness, which relates to the flow stress via a constraint factor of about three [11]:

(4) $$H_{\mathrm{Tabor}}\approx 3\;\sigma$$

For a material undergoing strain hardening a characteristic stress must be chosen, which can be approximated by the flow stress at a representative strain level of 8%: *H*_Tabor_ *=* 3 *σ*(*ϵ* = 8%). This implies neglecting the ISE and identifying *H*_Tabor_ with the micro-hardness level *H*_0_ approached for the largest nanoindentation depths (here at *F*_max_ = 100 mN)_._

Table 4 presents tensile test data in terms of the yield stress, the ultimate tensile strength and the final strength of the material, alongside the hardness predicted from the final strength, according to Tabor’s Equation (4). Here the final strength is defined as the force at rupture divided by the rupture cross-section as measured after the test. As shown in Figure 3 the average hardness at 100 mN for the as-received conditions is ~1250 MPa and ~2000 MPa for the pre-strained conditions, whereas Tabor’s prediction from *σ*_FS_ would result in average hardness at 100 mN of ~738 MPa for as-received, and ~1089 MPa for pre-strained iron, i.e., much lower values by almost a factor of two. This significant and systematic mismatch between the actually measured and the predicted hardness values is attributed to the pronounced pile-up formation during indentation [21,22]. This is even more significant if one notes that the Hall-Petch contribution to the flow stress (affecting tensile strength but not hardness) was not taken into account, when the predicted strength was calculated [13]. There is also a strong discrepancy of the apparent hardness increase due to pre-straining of about 750 MPa on the one hand, and Tabor’s prediction of the hardness increase of 3 Δ*σ* = 3 (*σ*_FS_ − *σ*_y_) which amounts to approx. only 300 to 670 MPa [11].

### 3.4. Elastic Modulus Correction

The indentation hardness results observed for unalloyed Fe exhibit a large systematic mismatch with the tensile data that affects both absolute strength and strength increase due to strain hardening (Figure 2 and Figure 3, and Table 4). The cause for this discrepancy does not imply inadequacy of the underlying Tabor constraint relation (4) in the first place, but can be attributed to the pronounced pile-up emergence, in particular, after pre-straining. This has been confirmed by AFM (Figure 5 and Figure 6). The mismatch between observed and predicted strength data can largely be reconciled by the application of an elastic modulus correction (EMC) procedure [24].

Assuming for the reduced plane strain modulus a reference value *E*_r,ref_ = 183 GPa,

(5) $$\frac{1}{E_{r,\mathrm{ref}}}=\frac{1-\nu_{s}^{2}}{E_{s}}+\;\frac{1\;-\;\nu_{i}^{2}}{E_{i}}$$

which is calculated using tensile data for a Young’s modulus *E*_s_ = 200 GPa and *ν*_s_ = 0.3, instead of *E*_IT_, Equation (3) from nanoindentation, and taking into account Equations (1) and (2) any micro-hardness bias deriving from systematic errors in the area function can be corrected by:

(6) $$H_{0,\mathrm{corr}}=\frac{H_{0}}{{\left(\frac{E_{r}\left(h_{c}\right)}{E_{r,\mathrm{ref}}}\right)}^{2}}$$

**Figure 8:**
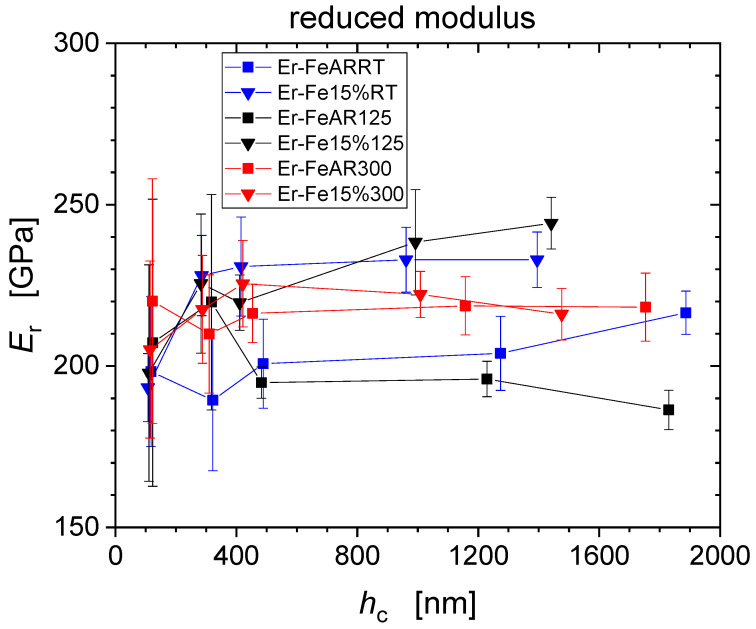
Reduced modulus values obtained from nanoindentation measurements and used for EMC procedure.

Figure 8 shows the reduced elastic modulus of the material corresponding to the indentation moduli presented previously in Figure 4. By definition, reduced modulus values are lower as compared to the indentation modulus, and range for the as-received material from ~190 GPa to ~220 GPa and for the pre-strained conditions from ~220 GPa up to ~250 GPa.

Table 5 compiles average reduced modulus values obtained from averaging the moduli for the three largest force levels 10 mN; 50 mN; 100 mN. These values have been used for the definition of common elastic modulus correction factors of each testing condition of the material.

**Figure 9:**
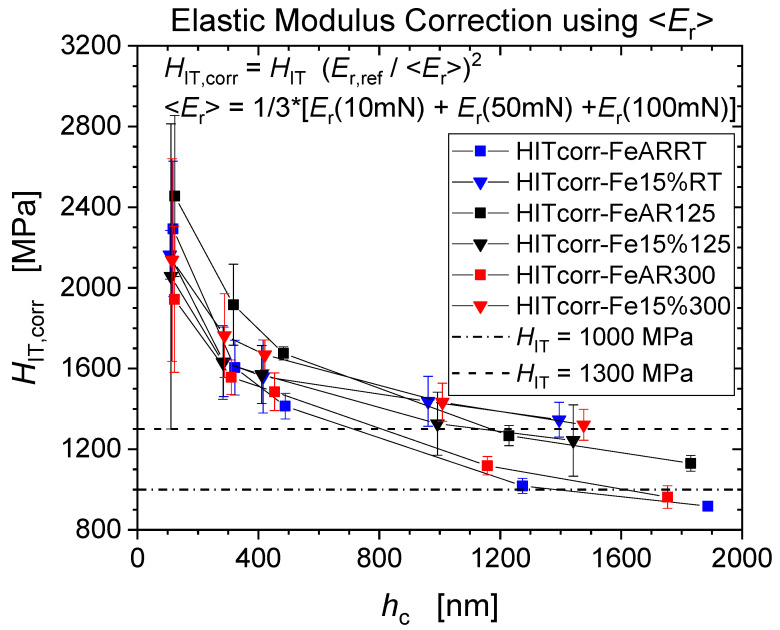
Corrected indentation hardness of un-strained and 15% pre-strained Fe.

The hardness values after application of the EMC procedure are presented in Figure 9 showing that now micro-hardness values level out at about 1000 MPa and 1300 MPa for the as-received conditions and pre-strained conditions, which is to be compared to the original apparent hardness levels amounting to 1250 MPa and 2000 MPa, respectively. In particular, we note that the EMC procedure results in a much smaller hardness difference between the as-received and pre-deformed materials conditions (~300 MPa), in closer correspondence with Tabor’s prediction (Table 4).

**Figure 10:**
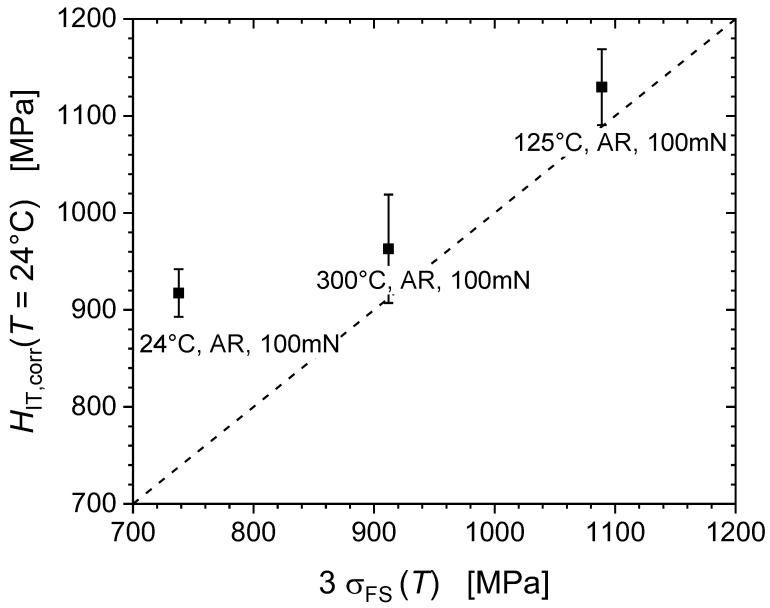
Hardness vs. tensile strength correlation after EMC.

Figure 10 demonstrates an inverse temperature dependence of the strength, both in terms of hardness and final tensile strength, the origin of which is discussed in the following subsection. After application of the EMC a reasonable agreement between hardness and tensile strength values has been accomplished, with the corrected hardness values being again much closer to the strength predicted by Tabor’s Equation (4).

### 3.5. Dynamic Strain Ageing (DSA) Effect on Strain Hardening

Figure 9 shows that strain hardening due to tensile pre-deformation is captured by the EMC corrected hardness measurements. The microhardness observed at the largest depths amounts to ~1300 MPa vs. ~1000 MPa in the as-received condition. The ISE is similarly pronounced for both conditions. Hardness differences are mainly due to dislocation multiplication induced during plastic pre-deformation and described by the Taylor contribution to the flow stress associated with forest hardening:

(7) $$\sigma_{f}=\tilde{\alpha }\mu b\sqrt{\rho }$$

where *μ* denotes the shear modulus, *b* the magnitude of the Burgers vector, *ρ* the dislocation density, and $\tilde{\alpha }=M\alpha$ a numerical coefficient which contains a factor α≈0.3 and the Taylor factor M≈3 and which is assumed to amount to 0.9.

Table 6 compiles the dislocation densities determined from TEM analysis and the corresponding Taylor stresses from Equation (7), as compared to the work hardening presented in Table 4, alongside the hardness increase (corrected by EMC). The hardness increase is more pronounced for the lower pre-straining temperature, as less dynamic recovery is occurring during plastic deformation. At low temperatures, mobile dislocations interact with quasi-sessile carbon impurities in interstitial solution with the otherwise high purity iron giving rise to additional solid solution strengthening of the material [25].

**Figure 11:**
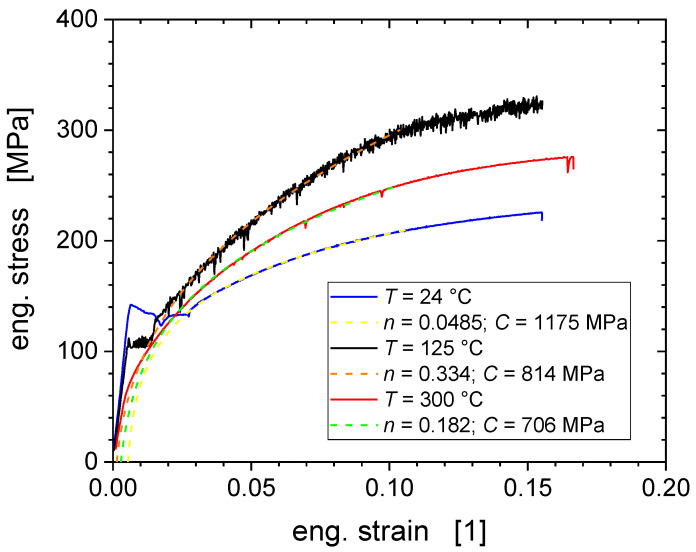
Stress-strain curves from tensile tests exhibiting serrated yielding at 125 °C and Lüders band propagation at RT and 125 °C. The dashed curves represent power law hardening rules *σ* = *C*(*ε − ε*_0_)*^n^* with hardening exponents *n,* strength coefficients *C*, and offset strains *ε*_0_ as reported in the legend.

The Fe-AR-125 °C aged (not deformed) material appears to be the hardest one, the increase of the corrected hardness being smallest, as during thermal ageing at the intermediate temperature of 125 °C dislocation cores can saturate with those carbon interstitials [15]. *H*_IT,corr_ ~1357 MPa at 125 °C represents the highest hardness value reported in Figure 10. At this intermediate temperature, dynamic strain ageing (DSA) occurs by the dynamic interaction of mobile dislocations and carbon interstitial atoms, noting that C concentration well below the present detection limit of about 300 ppm are sufficient to induce DSA [15]. DSA is associated with serrated flow (Portevin−Le Chatelier effect) which is clearly visible on the 125 °C tensile stress-strain curve in Figure 11. In body-centred-cubic (bcc) iron, DSA and serrated flow are associated with the so-called “blue-brittleness” temperature range of 100 to 300 °C [26,27,28,29]. The concomitant plastic instability and strain localization cause the hardness spread to increase most significantly through pre-deformation at the intermediate temperature of 125 °C, as illustrated by Figure 2 and Figure 3.

The tensile tests at RT and 125 °C also reveal yield phenomena at the onset of plastic deformation, i.e., inhomogeneous deformation (localized flow) through propagation of a Lüders band upon unpinning of the dislocations from their saturated solute clouds. The absence of serrated yielding and Lüders band formation for 300 °C shows that for the highest tensile deformation temperature the ageing does not matter as the solute atoms are sufficiently mobile to diffuse together with moving dislocations not letting their detachments to occur.

**Figure 12:**
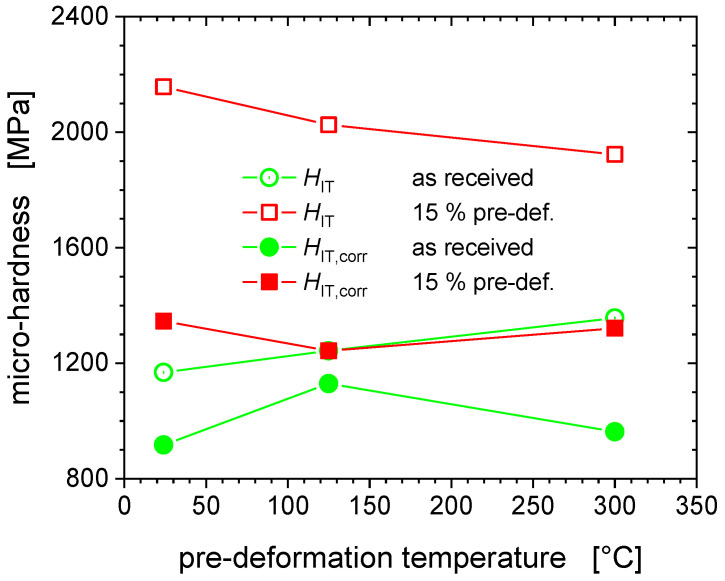
Hardness as a function of the pre-deformation temperature.

With respect to the hardening behaviour, we note that the flow stress and the strain hardening rate are both maximal at the intermediate temperature of 125 °C (Figure 11 and Figure 12) [15]. This is also reflected by a maximum of the hardening exponent (*n* = 0.334 for 125 °C), as indicated in the legend of Figure 11. These results are consistent with the microstructural observations and dislocation analyses reported for the different deformation temperatures. Dislocations appear to be heterogeneously distributed with regions of high density with pile-ups and tangles, separated by regions of low dislocation density (of 10^12^ m^−2^), which is comparable to the dislocation density in the non-deformed material. The highest dislocation density of 3.7 × 10^14^ m^−2^ was obtained at the deformation temperature of 125 °C, exceeding those at RT and 300 °C by factors of about two and four, respectively.

As regards the pre-deformed materials Fe-15%-125 °C (Figure 9) we observed *H*_IT,corr_ to exceed by only ~100 MPa the non-deformed reference level at this temperature, i.e., the lowest increase of the three cases (24 °C: Δ*H*_IT,corr_ ~ 430 MPa; 300 °C: Δ*H*_IT,corr_ ~ 360 MPa). The hardening potential is the lowest for 125 °C, as the material has already hardened significantly and dislocation density is saturated, and therefore their multiplication is rendered difficult [13].

The additional hardening associated with 15% pre-deformation and observed by the EMC corrected *H*_IT,corr_ at room temperature, possesses a minimum value for the intermediate pre-deformation temperature *T* = 125 °C, when DSA is most pronounced as shown in Figure 11. The efficient DSA at this intermediate temperature leads to reduced dynamic recovery and enhanced multiplication and accumulation of dislocations. Consequently, the work hardening rate during the tensile test is largest at 125 °C with *n* = 0.334 (Figure 11).

## 4. Conclusions

Hardness data from nanoindentation tests of unalloyed iron have been compared with bulk mechanical properties from tensile testing to 15% of strain performed at room temperature, 125 °C and 300 °C. The nanoindentation was carried out on reference and pre-strained material. A significant mismatch between the nanohardness and the tensile test results has been observed, attributed to the pile-up formation and subsequently required the nanoindentation results to be corrected accordingly. This has been accomplished by applying an elastic modulus correction (EMC). While in recent previous work the EMC served the correction of various sources of biases of data from multiple sources (round robin testing) [24], here it was specifically performed to account for changes in pile-up height induced by pre-deformation.

In doing so this work has improved our understanding of the way how nanoindentation testing can be used for mechanical property assessment in the presence of the intensive pile-up formation. Pronounced pile-up emergence, which became even more noticeable after the tensile pre-straining, was confirmed by AFM as cause of the large and systematic mismatch between the hardness data and values of the yield stress, the ultimate tensile strength and the final strength of the material as predicted by Tabor’s equation on the other hand. Consequently, it turned out to be possible to reconcile the difference between nanomechanical and bulk properties of unalloyed Fe by the elastic modulus correction. The EMC proved an effective alternative to correcting pile-up behaviour by AFM measurements of actual pile-up heights. At the same time, it is much less demanding.

Strain hardening behaviour and nanoindentation hardness results were found to be affected by the dynamic strain ageing (DSA) due to the presence of mobile C interstitials. The results evidence that the material aged at 125 °C is the hardest, as during thermal ageing dislocation lines saturate with interstitial carbon atoms with the consequence that the corrected hardness has a maximum at this temperature. This is also the reason why tensile tests reveal inhomogeneous deformation through a yield phenomenon (localized flow through propagation of a Lüders bands) and serrated flow due to dynamic strain ageing at the intermediate pre-deformation temperature of 125 °C.

## Figures and Tables

**Table 1:** Chemical composition of Fe (G379) and detection limits of impurities using SS-OES and ICP-OES, in wt.%.

Cr	Ni	P	Al	Si	V	W	Cu
0.002	0.007	0.003	0.023	0.001	<0.0109	<0.0099	<0.0091
**Mo**	**Co**	**C**	**Nb**	**Ti**	**As**	**Sn**	
<0.0082	<0.0080	<0.0067	<0.0036	<0.0020	<0.0012	<0.0010	

**Table 2:** Samples for nanoindentation and AFM from tensile specimens.

Specimen Name	Tensile Specimen ID	Temperature	Pre-Deformation
Fe-AR-RT	1	RT (24 °C)	Non-deformed
Fe-15%-RT	2	RT (24 °C)	15%
Fe-AR-125	3	125 °C	Non-deformed
Fe-15%-125	4	125 °C	15%
Fe-AR-300	5	300 °C	Non-deformed
Fe-15%-300	6	300 °C	15%

**Table 3:** Surface preparation sequence of G379 for nanoindentation.

Cloth	Suspension	Force [N]	Rotation [rpm]	Time [min]
Sic foil	Water	80	300/150 Co-rotation	3
MD-Largo	DiaPro All	120	150/150 Co-rotation	5
MD-Dac	DiaPro Dac 3	90	150/150 Co-rotation	5
MD-Nap	DiaPro Nap 1	60	150/150 Counter-rotation	3
MD-Chem	OPS	40	150/150 Counter-rotation	2.5

**Table 4:** Tensile properties from tensile pre-deformation tests and corresponding hardness according to Tabor’s predictions.

Deformation Temperature [°C]	Upper Yield Stress [MPa]	Lower Yield Stress *σ*_y_ [MPa]	Ultimate Tensile Strength [MPa]	Final Strength *σ*_FS_ [MPa]	3 *σ*_FS_ [MPa]	Δ*σ* = (*σ*_FS_ − *σ*_y_) [MPa]
24 (RT)	145	125	226	246	738	101
125	115	103	325	363	1089	222
300	82		276	304	912	194

**Table 5:** Averaged reduced modulus for 10 mN; 50 mN and 100 mN force, as used for EMC.

Condition	Reduced Modulus at 10 mN [GPa]	Reduced Modulus at 50 mN [GPa]	Reduced Modulus at 100 mN [GPa]	Average Reduced Modulus [GPa]
24 °C AR	201	204	216	207
24 °C 15%	231	233	233	232
125 °C AR	195	196	186	192
125 °C 15%	220	238	244	234
300 °C AR	216	219	218	218
300 °C 15%	226	222	216	221

**Table 6:** Dislocation densities from TEM observations and Taylor stresses from the corresponding dislocation forests, as compared to the work hardening (Table 4) and hardness increase (corrected by EMC).

Deformation Temperature [°C]	*ρ* [10^14^ m^−2^]	$\sigma_{f}=\tilde{\alpha }\mu b\sqrt{\rho }$ [MPa]	*σ_f_−* Δ*σ* [MPa]	Δ*H*_corr_/3 [MPa]
24 (RT)	2	255	134	143
125	3.7	346	86	38
300	0.8	161	−61	119

## Data Availability

The data presented in this study are available on request from the corresponding author.
